# “Attitude is the fifth delay”: perspectives of obstetric near-miss survivors and health care professionals on continuity and coordination of maternal care

**DOI:** 10.1186/s12913-025-12341-4

**Published:** 2025-02-19

**Authors:** Samuel M. Mulongo, Doreen Kaura, Bob Mash

**Affiliations:** 1 Department of Nursing, Faculty of Medicine and Health Sciences, Stellenbosch University, Cape Town, South Africa; 2 Department of Family and Emergency Medicine, Faculty of Medicine and Health Sciences, Stellenbosch University, Cape Town, South Africa

**Keywords:** Longitudinal continuity, Relational continuity, Parallel coordination, Sequential coordination, Obstetric near miss

## Abstract

**Background:**

Fragmentation in maternal healthcare contributes to preventable maternal deaths. Improving continuity and coordination can mitigate this issue by influencing interactions among providers, interdisciplinary teams, and patient-provider relationships. The obstetric near-miss approach has the potential to offer insights into continuity and coordination in maternal health. The aim of this study was to explore the perspectives of near-miss survivors and health care professionals on continuity and coordination of care in a primary health care system in Uasin Ngishu region, Kenya.

**Methods:**

This was a descriptive qualitative single case study. Data was collected through individual interviews, focus group discussions, and observation.

**Results:**

Four deductively identified themes emerged: *sequential coordination, continuity, parallel coordination, and access.* Sequential coordination was influenced by antagonistic relationships between healthcare providers in primary care facilities and the main referral hospital. Longitudinal and interpersonal continuity were driven by positive interactions between women and healthcare providers, characterized by availability, willingness to listen, and addressing personal issues. Parallel coordination was affected by heavy workloads and ineffective task shifting within primary care facilities. Finally, access-related issues included accommodation strategies such as telephone hotlines for pregnant women, out-of-pocket payments for specialist consultation, and alternative referral pathways different from the formal pathways in the health system.

**Conclusion:**

To promote continuity and coordination in primary health care for pregnant women, there is a need to focus on social competencies and skills among health care professionals providing obstetric care, encourage accommodation strategies in facilities, reduce or eliminate out-of-pocket payments for referred women, address attitude and professional conflicts among midwives at different levels in the referral pathway and address workloads and task shifting in primary care facilities.

**Supplementary Information:**

The online version contains supplementary material available at 10.1186/s12913-025-12341-4.

## Plain language summary

**What was the aim of this study?:**

To reduce the chances of mothers dying during childbirth, healthcare providers need to actively manage specific complications such as bleeding after birth, pre-eclampsia, eclampsia, difficult labor, preterm labor, and newborn infections. However, dealing with these priority issues will not work well if different healthcare providers, levels of care and teams of experts do not work together. This involves promoting therapeutic relationships between pregnant women and their healthcare providers (interpersonal continuity) and making sure that a pregnant woman visits the same provider across pregnancy (longitudinal continuity). With regard to health providers who attend to pregnant women, they should collaborate effectively within the same facility, for example, during emergencies (parallel coordination) as well as during referral (sequential coordination).

In this study, we interviewed women who had a near-death experience during pregnancy, labor, or delivery to gain insights into how their relationship with healthcare providers and antenatal visitation practices may have influenced their near-death experience. Likewise, we interviewed healthcare providers in health centers to gather information on how the coordination (or lack thereof) in their care could contribute to women experiencing a near-death event.

**What are the main findings of this study?:**

Women reported that they were more satisfied when health providers were available, listened to them and addressed their personal concerns. However, this was only possible if the woman visited the same provider over time. Another theme we identified is sequential coordination, or how well different healthcare providers collaborated in a step-by-step manner. We found that relationships between healthcare providers in primary care facilities and the main referral hospital was not always cooperative and sometimes even adversarial – which led to delays and ineffective management of emergencies. The way tasks are distributed and managed within healthcare facilities has an implication on coordination of care. For example, when midwives delegate duties to non-professional staff such as cleaners. Strategies such as telephone hotlines were instrumental for maintaining contact between the providers and pregnant women (longitudinal continuity). Other findings such as out-of-pocket payments for specialist consultations, and the exploration of alternative referral pathways outside the formal channels in the health system promoted continuity and coordination.

**What are the implications for practice?:**

Adopting models that promote therapeutic relationships between women and their providers may increase women’s satisfaction with care during pregnancy. Midwifery-led continuity models, as well as group antenatal visits, have been proposed. There is a need to address professional conflicts among midwives working at various levels in the referral system so that emergencies can be attended to quickly and effectively. Implementing strategies to make facilities more accommodating, such as flexible scheduling or toll free hotlines can keep women in constant touch with their providers. Within lower level facilities, finding ways to manage the workload efficiently and ensuring that tasks are appropriately distributed among healthcare professionals will promote coordination.  In lower middle-income countries such as Kenya, reducing or eliminating out-of-pocket payments for women referred to other healthcare facilities can improve access to necessary services and prevent complications that arise during labor and birth.

**Supplementary Information:**

The online version contains supplementary material available at 10.1186/s12913-025-12341-4.

## Background

Priorities for reducing maternal mortality towards achieving Sustainable Development Goals (SDGs) include essential childbirth care and management of postpartum haemorrhage, pre-eclampsia and eclampsia, difficult labour, preterm labour, and new-born infections [1]. However, addressing these priority issues cannot succeed in a fragmented health system [2]. As a result, there is a renewed focus on continuity and coordination of maternal health services [3]. Continuity and coordination are shaped by interactions among multiple providers, interdisciplinary teams, and patient-provider relationships across sectors and health facilities. They are among the five core principles of high-quality primary health care and have the potential to reduce fragmentation, improve utilization of health services, save costs and improve health systems performance [4]. These concepts are critical in settings like Kenya where the devolution of health services from national to county level may increase the fragmentation in maternal healthcare delivery. While devolved units received more resources, which can support improved infrastructure, the lack of coordination and changed behavior in health facilities may impede optimal maternal outcomes. Further, more levels of service provision require increased integration in communication between service points. Recent changes in Kenya’s maternal healthcare system require pregnant women to begin antenatal care at lower-level facilities, with referrals to higher-level hospitals only if complications occur. While this aims to decentralize care, it emphasizes the need for better coordination and continuity to ensure women receive timely and appropriate care.

For example, a recent study in Nairobi, Kenya suggested that pregnant women in are now likely to visit up to three facilities on average during pregnancy, perhaps in search of better quality care [5]. Recently, the World Health Organization (WHO) published a practice brief to enhance research and practice concerning these two essential concepts [6]. The various dimensions of continuity and coordination are summarised in Table 1.

**Table 1:** Dimensions of continuity and coordination [6]

*Construct*	*Definition*
Longitudinal continuity	The degree to which a client attends the same provider over time
Relational continuity	Entails the quality of the relationship between a client and their provider
Informational continuity	Availability of a client’s medical information at every visit over time
Sequential coordination	Collaboration across facilities or levels of care
Parallel coordination	Collaboration within facilities or the same level of care

The near-miss approach, which investigates women who survive severe morbidity during pregnancy, childbirth, and up to 42 days postpartum, is an established method for studying quality of care for pregnant women. In previous qualitative studies in South America, Nigeria and Uganda, near-miss survivors gave accounts of their experiences and perspectives on the quality of care across antenatal, intrapartum and postpartum care. They mentioned the need for more information, physical and human resources, and the need for more respectful interactions with nurses and midwives [7, 8]. In Kenya, near-miss survivors identified several challenges including inadequate birth planning, delays in identifying a suitable place for delivery, and delays in reaching the hospital due to distance, financial barriers or lack of emergency services [9]. However, these studies were all hospital-based, which may reduce women’s chances of criticizing facilities. Also, most of the studies explored women’s experiences only during the actual emergency but not from pre-conception and during antenatal care. A near-miss experience is likely a result of a chain of events and circumstances across different points prior to the experience itself.

The current study addressed some of the limitations in previous studies by focusing on near-miss survivors in their homes or in less threatening places. It also focuses on their journey from pre-conception to the actual near-miss and into the postpartum period in order to elicit distal factors. Finally, this study builds on previous studies by mapping the perspectives of near-miss survivors and health care providers onto the various dimensions of continuity and coordination (Table 1). Near miss survivors can enhance our understanding of how their relationships with health care providers (relational continuity) and antenatal care visitation patterns (longitudinal continuity) influenced their outcomes.

### Conceptual framework

**Fig. 1:**
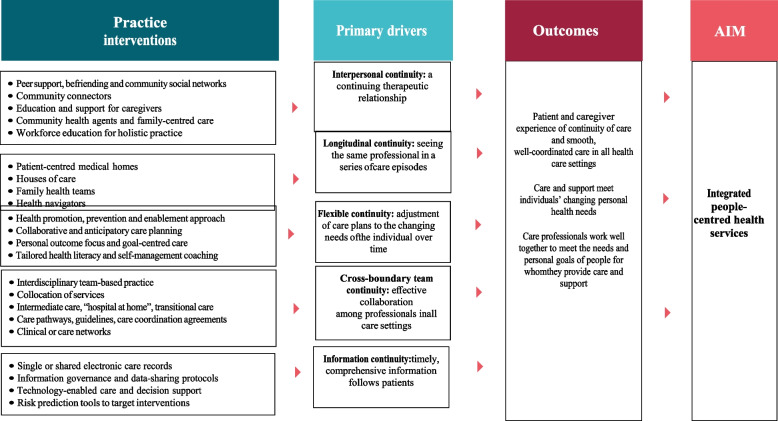
Drivers of continuity and coordination

Figure 1 below presents the drivers of continuity and coordination as depicted by the WHO operational framework for primary health care [2]. The framework depicts practice interventions that can improve continuity and coordination of care in primary care. Although most of the practice interventions are derived from research in chronic disease care, they are applicable to maternal health. The coordination of care, whether sequential or parallel, is influenced by the clinical activities at different touchpoints, and the continuity of care is influenced by interpersonal, longitudinal, management, and informational continuity. Failure of care coordination and continuity leads to delays in seeking care and poor obstetric outcomes, such as near-miss cases.

## Methods

### Study design

This article is part of a mixed-methods study assessing coordination and continuity in maternal healthcare using the near-miss approach. A quantitative phase determined the prevalence and determinants of obstetric near-miss in Kenya’s Uasin Gishu region. The qualitative phase, presented here, is an exploratory case study that investigates perspectives of near-miss survivors and healthcare professionals on continuity and coordination of care.

### Study setting

The study took place in the Rift Valley region of Kenya, focusing on the Moi Teaching Referral Hospital (MTRH) and its referral pathway. MTRH is part of Kenya’s tiered maternal healthcare system and serves as a national referral hospital. It is responsible for handling obstetric emergencies from around 250 lower-level health facilities across 20 administrative regions. Obstetric near-miss cases in this study came from Level 2 (dispensaries), Level 3 (health centers, maternity, nursing homes), and Level 4 (sub-county hospitals). Levels 2 and 3, provide Basic Emergency Obstetric Care (BEMOC). High-risk cases are referred to Level 4 or 5 hospitals, which offer Comprehensive Emergency Obstetric Care, including caesarean sections and blood transfusions. Emergency transfers rely on ambulance availability, fuel, and the readiness of the receiving facility.

### Study population

The study population included near-miss survivors and healthcare providers within the catchment area of MTRH in Kenya’s Uasin Gishu region. The near miss survivors were identified through obstetric records at MTRH, which contain detailed information on severe morbidities, interventions and the primary facility from which patients were referred. The population of near miss survivors was derived from the quantitative phase of the study published elsewhere [10]. In the quantitative phase, the maternal near miss ratio was determined to be 8.7 per 1000 live births. The most prevalent direct factors were severe post-partum haemorrhage (35%), eclampsia (18.9%) severe pre-eclampsia (17.4%), blood transfusion (79%), and hepatic dysfunction (3.7%).

### Selection of participants and study size

**Fig. 2:**
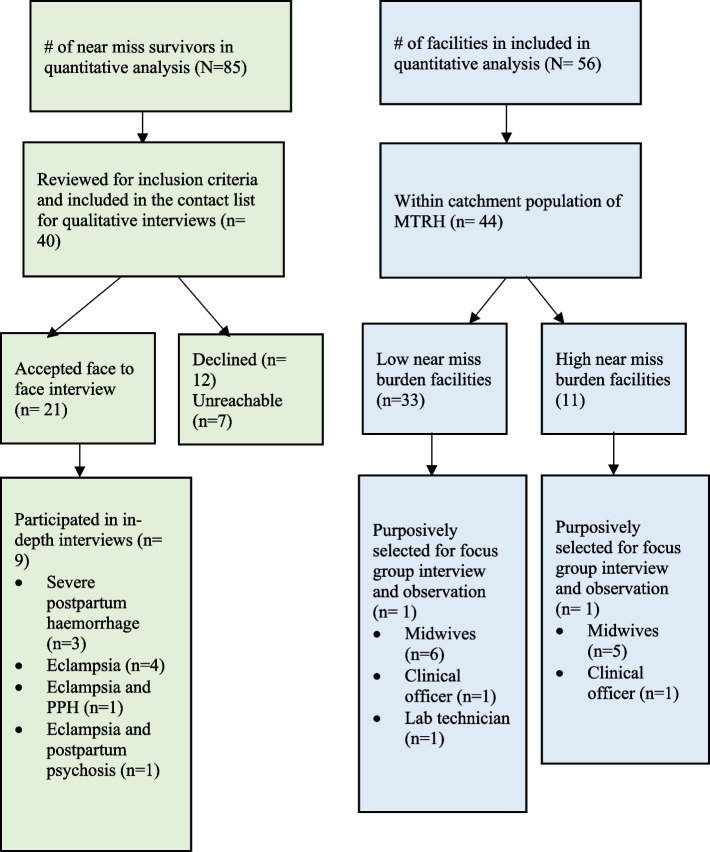
Selection of near miss survivors and facilities

Selection of participants for the study is summarized in Fig. 2 below. Out of 85 near-miss survivors from the quantitative phase, we included 40 in the contact list for qualitative interviews since they were within reasonable geographic distance making it feasible for the research team to reach them for interviews. Twenty-one agreed to face-to-face interviews, 12 declined, and seven were unreachable by phone. In the end, we interviewed nine participants: three with severe postpartum hemorrhage; four with severe PPH and eclampsia; and one with severe PPH and postpartum psychosis. Out of 56 facilities chosen for quantitative analysis, 44 were within the catchment population of the referral hospital. Among them, 33 had a low near-miss burden, and 11 had a high near-miss burden. We selected one facility from each region and formed focus groups. The focus group for the low-burden region included six midwives, one clinical officers, and one lab technician. In contrast, the focus group for the high near-miss burden region comprised five midwives and one clinical officer.

### Data collection

Interview guides were guided by the WHO conceptual framework on continuity and coordination in primary care [6].

#### Individual interviews with near miss survivors

The interview guide (supplementary file) was designed to gather the following information from near miss survivors: (1) their perspectives of the journey through pregnancy, birth or postpartum; (2) perspective during the emergency management of eclampsia and postpartum haemorrhage; (3) perspectives on their relationships with health care providers during pregnancy, birth and postpartum and how it may have influenced their near-miss situation; and (4) their perception of coordination of the care that they received and how it may have influenced the near-miss situation. The researcher then piloted the interview guide with two women from the study site. The pilot data were included in the study as no changes were made to the interview guide.

Individual interviews took place at the woman’s place of choice, though most participants chose to do the interviews in their homes. The principal researcher (SM) conducted all the interviews. Participants were debriefed on the purpose and nature of the study, after which they were asked to provide informed consent. Since all participants understood either Kiswahili or English, no translation was needed. The researcher audiotaped the interviews with the participant’s permission during all the interview sessions. Most discussions took between 30 to 40 min. The interviewer continuously monitored theoretical saturation which he deemed to have occurred when there was no new information after the 8th interview. The 9th interview confirmed saturation.

#### Focus group discussions

Focus group discussion guides (supplementary file) were developed to assess healthcare providers’ perspectives on continuity and coordination of care components that influenced near-miss situations. Questions focused on managing eclampsia and postpartum haemorrhage-the main morbidities underlying near-misses-and relationships with women during antenatal, intrapartum, and postnatal care. Probing questions explored interpersonal and relational continuity, as well as care coordination within primary level care-and between primary healthcare and referral hospital in order to identify how they influenced near-miss occurrences.

The principal researcher conducted two focus group discussions-one at HC-A (a High near-miss burden facility) and one at HC-B (a low near-miss burden facility). The selection of HC-A and HC-B as study sites was based on the results of the quantitative phase of the study, which identified regional differences in occurrence of severe maternal morbidity [10]. Specifically, mothers from facilities in the Western region were found to be more likely to present with severe morbidity compared to mothers from other regions (*p* = 0.016) [10]. This influenced the selection of HC-A as a facility with a high near-miss burden. In contrast, HC-B, located on the Eastern region, was selected as a facility with a lower near-miss burden, based on its relatively lower rates of severe morbidity. The assignment of “high” or “low” near-miss status to these facilities was thus informed by the regional trends in maternal health outcomes observed in the quantitative phase, with facilities showing higher rates of severe morbidity categorized as high near-miss burden, and those with lower morbidity rates categorized as low near-miss burden.

Although midwives and clinical officers participated in the same focus group discussion, each was given ample time to discuss issues based on their professional perspectives. The English language was used for communication, although participants were allowed to express their views in Swahili if necessary. A dedicated audio-recorder was used to record all focus group discussions. To ensure trust-building, the researcher clarified the purpose of the focus group and maintained an appropriate physical and non-threatening environment. To compensate for their time, each participant received 1,000 Kenya Shillings (equivalent to USD 10.) The discussions lasted approximately two hours each and were held in a private room within the health centers to provide a comfortable and confidential setting.

#### Observation

The researcher conducted participant observation for two weeks, one week at HC-B (low near miss burden) and one week at HC-A (high near miss burden). An observation checklist was used for participant observation. The observation guide used was semi-structured, which allowed the researcher to write detailed notes based on what was observed. This checklist focused on elements of day to day maternal health care related to the main concepts in the study. These included observing how much time midwives spent with pregnant women during antenatal visits and whether they allowed enough time for the women to share their concerns about the pregnancy. For coordination, the researcher observed how different healthcare workers-such as midwives, nurses, and clinical officers-collaborated, particularly in handling emergencies and transferring patients to MTRH. Although a true emergency was observed only at HC-B (low near miss burden facility), it provided valuable insights into the coordination process. Finally, the researcher noted the tools and resources available at each health centre, considering how these might influence care delivery. The researcher also observed aspects like patient admissions, handovers, referrals, and communication between facilities. The observation process was flexible and iterative: insights from focus group discussions helped guide further observations, and observations informed the discussions in turn.

The researcher observed the actors, activities, and means of communication that enabled continuity and parallel coordination in HC-A and HC-B. The researcher used the “observer as participant” approach, which enabled a close interaction with health providers without playing an active role in core activities. Field notes, photos, and records of informal conversations were used for data capture.

### Data management and analysis

Focus group discussion and individual interview data were analysed together. The researcher chose this approach since it enhanced the interpretation of the context, as the group dynamics observed in the focus group discussion complemented the individual perspectives captured in the survivor interviews. Additionally, it became evident that keeping the data sources separate would not have been as effective, as integrating these differing viewpoints provided a more coherent and meaningful set of conclusions. The data analysis and interpretation followed the thematic analysis steps for qualitative data (Yin, 2018). *Step 1* involved reading transcripts line by line to determine general perceptions, noting common threads, words, and phrases. *In step 2*, data was coded by searching for unique opinions and perspectives to explore emerging patterns, using memos to document thoughts. Similar codes were merged before creating categories. *In step 3*, codes were grouped into categories, and themes were generated using the conceptual framework’s nomenclature, focusing on interpersonal continuity, longitudinal continuity, parallel coordination, and sequential coordination. Data from individual interviews, focus group discussions, and observations were triangulated to identify convergence or disagreements. Results were categorized into interpersonal, informational, or longitudinal continuity and parallel or sequential coordination. Actors, collective actions, and coordination means within and between healthcare facilities were noted. Themes from each data source were compared iteratively for convergence or dissonance, and all segments were evaluated for prominence and meaning. A matrix presented the triangulation results, and the emerging themes were confirmed through discussions with other authors.

### Reflexivity

In the process of developing the interview guide for this study, the researcher relied on experience as a university lecturer in nursing and community health, along with working in community health setups. This background positioned the researcher to ask relevant and contextual questions, considering the understanding of the local language and its cultural aspects, which is not far from the researcher’s home county. Efforts were made not to develop personal relationships with participants, though this was difficult given the emotional nature of the topic at hand.

While collecting the data, the researcher was conscious of the positionality occupied. It was understood that health workers might face certain challenges. Since the researcher had worked in similar situations and had an appreciation of structural challenges and pressures, there was often empathy with the participants. This made the effort to avoid introducing personal opinions about the answers, noticing that the researcher holds strong support for comprehensive policies to eliminate maternal morbidity, indeed strenuous. This neutrality was also maintained during the analysis of qualitative data and the writing of the report, where an effort was made to report the results of the research in the most accurate and unbiased way possible, to reflect what the participants said rather than imposing personal conclusions on them.

### Trustworthiness

Face-to-face member checking for all individual interview participants was not feasible because of the remote locations that some of the participants live. However, the researcher conducted follow up interviews by phone for three participants. Further, the researcher involved two experienced qualitative researchers, namely DKM and RM. The two researchers played an advisory role from the study design, design of instruments, data collection and data analysis. Throughout data analysis, periodic re-coding was carried out to refine the emerging themes, ensuring consistency and accuracy. Triangulation of data sources was used to verify findings. For example, when focus group participants in high near miss burden facilities mentioned limitations in resources, the researcher was able to verify this through observation.

## Results

### Description of participants

Participant characteristics are shown in Table 2 below. In summary, nine women with obstetric near miss participated in interviews. Ages ranged from 22 to over 30. Four women experienced eclampsia, three had severe postpartum haemorrhage, one had both eclampsia and postpartum hemaorrhage, and one had severe pre-eclampsia and postpartum psychosis. Eight delivered by caesarean section and were multiparous. All were married, with varying education levels. Healthcare professionals from two level 3 facilities participated in focus groups. HC-A (high near-miss burden) included six nurse-midwives and one clinical officer, while HC-B (low near-miss burden) had five nurse-midwives and one clinical officer.

**Table 2:** Participant characteristics

Characteristic	# of Participants	Percentage
**Near-miss survivors (interviews)**		
***Age***		
> 30 years	5	55.6%
25–30 years	3	33.3%
22 years	1	11.1%
***Obstetric ccondition***		
Eclampsia	4	44.4%
Severe Postpartum Haemorrhage	3	33.3%
Both Eclampsia & Postpartum Haemorrhage	1	11.1%
Severe Pre-eclampsia & Postpartum Psychosis	1	11.1%
***Delivery Method***		
Caesarean Section	8	88.9%
***Parity***		
Multiparous	8	88.9%
***Relationship Status***		
Married and Living with Partner	9	100%
***Education***		
Secondary School	4	44.4%
College-level	4	44.4%
Postgraduate (Masters)	1	11.1%
**Health Care Providers (Focus Groups)**		
***HC-A (high near-miss burden facility)***		
Nurse-midwives	6	75.0%
Clinical officer	1	12.5%
Laboratory technician	1	12.5%
***HC-B (low near-miss burden facility)***		
Nurse-midwives	5	80%
Clinical officer	1	20%

### Thematic overview

We *deductively* identified four themes based on the WHO framework for continuity and coordination. The most discussed theme was collaboration of health care providers and health teams across facilities, and we labelled this *sequential coordination.* The second theme related to relationships between health care teams and women across the continuum of care, and we marked this *continuity*. The third theme was collaboration between healthcare professionals and teams in primary healthcare settings, and we called this *parallel coordination.* Finally, other factors that did not fall into one of the categories of continuity and coordination but contributed to the overall outcomes were classified as *access.* Table 3 below shows emergent themes and subthemes.

**Table 3:** Emergent themes and subthemes

THEMES	SUB-THEMES
Sequential coordination	– Antagonistic professional relationships – Specialist consultation – Emergency services and transport – Information transfer
Parallel coordination	– Community-based follow up – Task shifting
Continuity	– Communication of diagnostic and clinical information – Woman centeredness
Access to services	– Telephone hotline as an accommodation strategy – Birth planning and companionship – Alternative referral pathways

### Theme one: sequential coordination

Sequential coordination emerged in this study as an outcome of the network of activities within the referral drainage. The activities within the primary health care setting include collective actions of the midwife, clinical officer and the community health volunteer, the means of communication, rules of engagement and the division of work. We categorized the subthemes that emerged from this theme as professional relationships (collective team actions), specialist consultation (individual action), emergency services and transport (tools of work), and information transfer (means of communication).

#### Subtheme one: antagonistic professional relationships

Collaboration between facilities and collective team actions during an emergency emerged as necessary for effective sequential coordination. Disagreements between professionals, specifically between midwives in referral and referring facilities, were evident. Consequently, near miss survivors felt that these disagreements delayed their care and management, as disclosed by the quotation below.

*…. In fact, the people at [facility X] really blamed [facility Y], saying that you should have brought this patient earlier, why did you wait until she worsened for you to make a phone call? You should have called early. Although when they realized things are tough, they [facility Y] switched off the phone, they no longer received phone calls… [near miss survivor - severe postpartum haemorrhage]*

Perceived power imbalances lead to mistrust between professionals, causing staff at lower levels to fear seeking guidance. Disagreements arise from suspicion about technical competence in managing emergencies and fear of blame for adverse events or maternal deaths. These issues are more common among women referred to multiple facilities before reaching the main referral hospital.

*At times attitude causes delay, that’s a fifth delay, attitude is a fifth delay, because you are from (PH-1), you have called, you reach there you are told this patient, now, is not even that serious., I had also a client with a cord presentation, I am being challenged is this a cord presentation or a cord prolapse? so the person had to do VE (Vaginal examination), in the process cord the cord ruptured, so you find that clients are not dying along the way, they are dying at (RH-B) and then the blame becomes political, the MCA (Member of county Assembly) calls the CEC (chief executive officer)…. the buck stops kwa ground …the buck stops with us in the referring facility [Midwife 01, HC-A, high near miss burden facility]*

The attitude of the health care professionals emerged as a delay for care as it influenced the mistrust and caused barriers in communication between the midwives in referring and referral facilities. Although the midwife from the referring facility was sure regarding her assessment of the client, the midwife at the referral hospital doubted the assessment. The mistrust caused the emergency to complicate the situation further.

#### Subtheme 2: failure to follow up on specialist referral

In both high and low near-miss burden facilities, the failure of mothers to follow through on their referrals to specialist consultation emerged as a potential determinant of the occurrence of near misses. Although the health care providers in the referring facility do their due diligence in referring the high-risk women for specialist care, the individual action of the woman influenced the outcome.

*Actually most of the clients even the pre-eclampsia clients , and the pregnancy induced clients, most of them we usually advise them to go to (RH-B), even the multiple pregnancy, even the breech but you find the situation whereby you are at night, the same client who, even if you check on the ANC book, the same client was referred to high risk clinic, but the same client comes during labour comes with complications[midwife 01, HC-A, high near miss burden facility]*

*Maybe I can say, it’s true, it applies to us also, sometimes if they are referred and they don’t go home, like those who are rhesus negative and that time we don’t have the anti-D, so we refer them to go for referral, but sometimes they don’t , they come now when they are in labour or second stage but they is nothing much we can do, but now after that now we have to refer them again to get the anti-D, so I don’t know, our mothers have a phobia of going to referral… [Midwife 03,HC-B, low near miss burden facility].*

Health care providers suggest that several factors contribute to women not attending specialist consultations at the main referral hospital. These factors include out-of-pocket fees for accessing specialists, travel costs due to the distance (e.g., 50 km from the high near-miss facility), and long waiting times for an obstetrician/gynaecologist on designated days. There is no mechanism in place to monitor whether these women have attended their referral appointments, which may exacerbate the issue. These findings are connected to the results discussed in the “community follow-up” section.

#### Subtheme 3: lack of emergency services/transport

A stark difference between high and low near-miss burden facilities was the availability of an ambulance. In the high near-miss facility, an ambulance was shared between several facilities and therefore was not readily available in an emergency. The lack of ambulance services points to the scarcity of investment in resources for this facility. Also, from the same focus group discussion, we learned that health care providers have learned to manage the situation through improvisation (see the quote below). Although most facilities with ambulance problems tend to devise ingenious workarounds, they tend to be counterproductive because they seem to “normalize” delays.

*After informing them is when we look for means of transport, we have a good referral system although we don’t have a vehicle here, we have one at Moi’s bridge health centre most of the time when we call them we are able to reach the ambulance and if it’s not available, we have a call centre at UGDH where they look for an ambulance from anywhere, any corner so long as they send an ambulance to us..[midwife 04, HC-B - low near miss burden facility].*

*There is one nurse at CWC who tells me she must rush to the maternity in case there is an emergency. I cannot see an ambulance; on inquiry I am told the facility does not have one – they call in case of an emergency. [Observation field notes from HC-B, high near burden miss facility]*

Therefore, emergency services were a barrier to swift care. The health care professionals would have required timely responses when referring a woman.

### Theme two: continuity

This theme was broadly related to how women perceived their relationships with health care providers, and how this relationship influenced their probability of returning to the same facility for subsequent consultations. The subthemes that emerged under this theme were woman-centeredness, miscommunication, and poor follow-up.

#### Subtheme one: woman centeredness

Women-centred care was highlighted as important to near miss survivors in their relationships with health care providers, particularly midwives providing antenatal services. Satisfaction increased when women received care from familiar health care providers, either from antenatal care or community connections. Women expected sensitive treatment even from unfamiliar health care providers, with perceived insensitivity causing them to change facilities. Sensitive care included proper palpation and attentively listening to concerns. Women valued health care providers who addressed their concerns, leading to increased trust and a willingness to return to the same provider. This factor contributed to how women assessed health care provider competency.

*For that facility, you lie on the bed, immediately they place that thing for listening, after they listen, they tell you that you are ok, please climb down. You see, maybe you have come with some issues, the way am telling you I was in so much pain and my legs, I wasn’t able to walk well. So, you want them to touch you, so that you know why the pain is there. So they didn’t do that ….so I went to facility (name), also they were not good. So, I went to another one, I can’t remember the name, it’s in the bush, the doctor was not bad, he was touching me…unfortunately they transferred him from there…so I stopped going there, I went to this one here (name). Here it was the same, they just listen, they just listen, they don’t palpate well. So, I said, let me go to this one (name), at least they seem to manoeuvre the baby, in fact they are the ones who told me how the baby was positioned, so I even preferred to deliver from there… [Near miss survivor - eclampsia]*

This woman changed facilities because she felt ‘not listened to’. The lack of follow-up care from one facility to the next caused the woman to have inadequate assessment and management during pregnancy and birth. Further, interpersonal care was necessary for the woman to feel confident in the care provided.

#### Subtheme two: miscommunication

How health care providers communicated examination findings and other clinical information seemed to influence women’s perception of the health care providers. Women interpreted terms such as “bad obstetric history” as stigmatization of their fertility. The perceived judgmental attitudes by health care providers seemed to erode informational, relational, and longitudinal continuity because women were unwilling to visit the clinic subsequently, as illustrated by the quote below.

*Normally when I went to hospital, when I give my history about like I am para 0 plus 4, they say eeh that’s interesting point. They will say what happened, until you are para 0 plus 4? So, they normally categorize me to be part of bad obstetric history.… Sometimes it is not good because my prayer is that my fertility will be good.... [Near miss survivor - eclampsia]*

The inadequate or inappropriate communication with women becomes a catalyst to disempowerment. The women do not necessarily understand what to expect, especially with previous poor experiences. The women felt desperate for positive outcomes, although the communication from midwives made them sad.

### Theme three: parallel coordination

This theme emerged as the collective activities within the primary health care setting that influenced care coordination. This involved how the midwives, clinical officers and community health volunteers acted as a cooperative team, enabling desired or undesired outcomes. The themes that emerged included: (1) inappropriate task shifting and (2) poor community follow-up.

### Subtheme one: inappropriate task shifting

Heavy workloads, staff shortages and inappropriate task-shifting emerged as the main detriment to parallel coordination. Improper task-shifting emerged as scenarios where, due to staff shortages, unqualified workers such as cleaning assistants performed monitoring of women in labour even when they had arrived in the facility early enough for standard management, as described below. The staffing norms for clinical staff in level 3 facilities are expected to be at least three nurses and one clinical officer at any given time. In the high near-miss facility, there were two nurses and one clinical officer. The situation worsened during night shifts as nurses perform both nursing and clinical duties, which may be burdensome.

*The person I blame is the nurse that I found there, instead of attending to me he left me saying that he has some other business. He left me with the person who cleans the ward. So, reaching 9 am another nurse came. He is the one who should have taken responsibility to say, “we can’t manage this patient, lets refer… [Near miss survivor – postpartum haemorrhage].*

*The challenges we have is shortage, many times we have only one person like a weekend, so imagine once the clinician has gone the nurse remains alone on the whole facility and am imagining now, suppose she gets an eclampsia or pre-eclampsia? … [midwife 02, HC-A, high near miss burden facility]*

While the women felt neglected, the midwives felt overworked due to staff shortages. The women viewed task-shifting to unqualified personnel such as cleaners as irresponsible and the reason for delayed referrals.

#### Subtheme 2: poor community follow up

Failure to follow up on high-risk women within the community and household levels emerged as detrimental to parallel coordination. In Kenya, community health volunteers and extension workers are mandated to conduct household visits for preventive and promotive care, which includes checking on any identified high-risk woman and reporting back to the facility. Near miss survivors consistently said that although some were known to have pre-eclampsia, they were neither followed up nor contacted by the facility. This may point to poor coordination between the health facility and community-based teams. Specifically, the health centre clinical team leader supervises community-based extension workers and volunteers.

*Ok you know what is happening , what we normally do is when we find a client who has an issue we normally take their number but we have never done any follow up to be frank, maybe we can start on that but we normally do is that when we see a situation that requires attention we exchange the numbers in case of anything we just call that is what we normally do… [midwife 05, HC-B, lower near miss burden facility]*

Some participants perceive this to have contributed to them experiencing a near miss. Nurses and midwives blamed the failure to follow up with women in the community on their job description, which does not include household visits. Thus, it seems unclear how household visits are organized and whether they are carried out.

### Theme four: access to services

This theme emerged, reflecting the need for service relationships that connect people to the activities within the health care setting. However, access was enabled or hampered through communication, health literacy, companionship, and alternate referral pathways.

#### Subtheme one: telephone hotline as an accommodation strategy

Accommodation emerged as a critical strategy for continuity of care. In this context, accommodation is defined as health systems interventions for increasing access through special consultation hours and other arrangements. We observed that in the low near-miss facility, there was a telephone hotline for pregnant women to call the facility (free of charge) in case of an emergency or at the onset of labour pains. In contrast, there was none in the high near-miss facility.

*It is now one hour since the emergency unfolded, and apparently the patient is stable…each of the rooms has a nurse. there is a toll-free number for pregnant mothers to call in case of an emergency [Observation notes, HC-B - Low Near miss burden facility]*

#### Subtheme two: health literacy

This subtheme emerged as the near-miss survivors’ own capacity to make decisions and act during pregnancy or around childbirth. These included empowering women with the appropriate sexual and reproductive health information for self-management and creating positive practice environments. The ability for women to identify potentially serious signs and symptoms during pregnancy emerged as a necessary measure to prevent near-miss events. Women’s empowerment before and during pregnancy emerged as an enabler to birth preparedness***.*** Participants reported engaging in downplaying or minimizing the significance of symptoms, particularly for pre-eclampsia and eclampsia. Part of the reason appeared to be driven by popular beliefs about specific symptoms considered “normal” during pregnancy.

*That day I had gone to work morning hours, but my body was swollen, the whole body including the face ok, I didn’t think it’s a big deal, at that particular time, I thought its normal because people say when a woman is pregnant her legs usually swell… [near miss survivor - eclampsia]*

Most participants reported attempts to manage their conditions at home based on cultural practices or family advice. These ranged from purchasing over-the-counter medications without prescriptions to using herbal medicines. This practice was more common among women who experienced pregnancy-induced hypertension.

*I just wake up, try my best, when they find that its high, I just take drugs…sometimes I take drugs and it refuses, so when it goes high, I take garlic so that it goes down. That is how life had become until now, in-fact I don’t take medicines anymore, I have ever gone to take medicines, when it goes high, I take garlic [Near miss survivor - eclampsia]*

### Subtheme three: birth planning and companionship

Birth planning and anticipation of steps to take emerged as necessary in supporting women through pregnancy, birth and postpartum, especially when they needed to make swift decisions. A common subtheme among participants was the lack of anticipation or preparedness for their day of delivery. This was the case even among mothers with a previous near-miss situation. For example, a mother who experienced severe postpartum haemorrhage said:

*The labour pains started at three in the morning, at that time it was not very painful, but I knew now my time for delivery has reached…because sometimes the abdomen has some other pain, I thought I should go to hospital because for my second child, I ignored the pain and ended up delivering by the roadside… [Near miss survivor - severe postpartum haemorrhage]*

Further partner support was vital for swift responses in cases of emergency. Sometimes, actions to be taken on the delivery day were entirely based on whether the husband was available to support the mother or if there was another form of support at home. The woman quoted below waited through her labour pains for her husband to return home before he took her to the hospital. She later experienced retained placenta and severe postpartum haemorrhage. She was briefly admitted to ICU in the main referral hospital and made a complete recovery.

*It started around midnight, so I persevered because my husband had not slept home that day, so around three in the morning is when I called him to come and take me to hospital, that’s when he came immediately and used a motorcycle to take me to (Facility name****)..**** [Near miss survivor - postpartum haemorrhage]*

Although this woman started bleeding at midnight, she could only be transported to the emergency unit three hours later using transportation that could worsen her bleeding.

#### Subtheme four: alternative referral pathways

Referral networks based on social capital were effective ways to find appropriate emergency services. Participants reported that sometimes referral was facilitated by a friend, spouse or someone in the community who “knew” a doctor or other health provider. Often these referrals appeared to lead to a faster consultation, admission, or treatment process.

*First, when I developed that bleeding when the bleeding started, my husband called [Doctors’ name]), the gynaecologist doctor at [facility name], and he asked him that we rush to hospital and when we reached there, he just managed [my bleeding] …[Near miss survivor, postpartum haemorrhage].*

Access to immediate consultation in case of an emergency was necessary for women. It emerged that it could be the reason this woman survived mortality, although she ended up with a near miss.

### Subtheme five: cost / financial barriers

Creating enabling environments emerged as necessary for the women to access the care they need. Managing socio-economic issues featured among near-miss survivors as a cause and consequence of their near-miss experience. Failure to return for check-ups or follow referral instructions was all attributed to a lack of money for bus fare or the need to do household chores as the sole breadwinner.

*So when I reached here, I knew there is trouble, bus fare for going and coming back... I just went once for check-up but the second time the problem was bus fare. Another problem is that I have young children, I did not have anyone to leave the children with, so I gave up, I have never gone back for check-ups****…**** [Near miss survivor, eclampsia]*

Within the Kenyan health care system, maternal care at lower-level facilities is entirely free of charge (subsidized). However, should a mother be referred to one of the major referral hospitals they are expected to pay an out-of-pocket fee to access specialized care. This fee also seems to dissuade mothers from seeking care after being referred from lower-level facilities. The quote below is from a nurse-midwife in a high near miss facility.

*… we had a patient with epilepsy , she was epileptic and they needed to, they [referral hospital] say, “you don’t bring that client unless you have money for CT scan”, and sometimes the clients we are handling sometimes they don’t have money at that time, so it delays the referrals that’s one of the challenges, they come with money so that when they reach there they go directly for CT scan… [midwife 05, low near miss burden facility]*

A client with epilepsy requiring an urgent CT scan could not receive it unless she had money to pay on arrival at the referral hospital. The delay in the investigation caused a delay in the care provided to the woman.

## Discussion

### Summary of findings

Woman-centred care, with sensitivity to women’s needs and non-stigmatizing language, supported interpersonal continuity. Accommodation through special hotlines and community follow-up improved longitudinal continuity by maintaining contact with health providers. Sequential coordination involved collaboration between facilities and provider power dynamics, while parallel coordination was influenced by task shifting and workload management. Access factors included women’s empowerment in reproductive health, family support, timely high-risk mother identification, and cost of care in referral facilities.

### Interpretation

#### Drivers of interpersonal and longitudinal continuity

In this study, women were more satisfied and likely to return for future consultations when health care providers addressed their concerns and committed more time to listening to them. This finding agrees with a previous systematic review which showed that women value a positive pregnancy experience that goes beyond routine clinical care and focuses on positive interpersonal behaviours [11]. Such behaviours include availability, willingness to listen, and addressing personal issues. A recent Cochrane review concluded that midwifery-led care models promote more interpersonal continuity than other models [12]. Midwifery education encourages practices such as natural birthing positions and less invasive procedures, which mothers may interpret as a less threatening environment.

Nevertheless, it cannot be assumed that midwives will automatically promote levels of interpersonal continuity expected by the woman. As was found in this study, multiple factors such as the physical clinic environment and workloads may compromise the time and space that the midwife commits to the woman in each consultation. It is also possible that health care providers do not consider interpersonal continuity important because of the complexity of linking it to perinatal outcomes.

#### Drivers of sequential coordination

In this study, antagonistic professional relationships between referring and referral facilities were common in high and low near-miss facilities. This study agrees with previous studies concerning collaboration between facilities during emergencies [13]. Generally, some level of disagreement is expected during referral, for example, when referrals are unannounced or when there is a lack of adequate referral information. However, a breakdown in collaboration generally has a detrimental effect on perinatal outcomes [14]. Most of the conflict reported in this study was between midwives-a useful finding because it sheds light on the less discussed problem of hierarchies, cadre stereotyping, and power dynamics among nurses and midwives.

EMOC training is designed to enhance teamwork and communication during an emergency [15–17], nevertheless, retention remains a significant issue. A recent systematic review found that communication skill retention decreased by 78% in the first three months after training [18]. Addressing this complex problem may require more innovative methods beyond the division of labour by emphasising integration and coordination [19]. A multipronged approach incorporating recent evidence from both high- and low-income countries is needed. Structured handover tools and closed-loop communication in teams have been suggested. There is also some evidence that professional antagonism is more pronounced during stress and panic. Therefore, situational awareness training should form the foundation of continuous training for midwives. This approach creates confidence in midwives’ ability to perform under stressful conditions. Diversification of training methods may also be warranted, given the different learning styles and professional orientations.

The lack of emergency transport services was a barrier to effective sequential coordination. This finding is similar to a qualitative study among near misses in Malindi region of Kenya [20]. In Kenya, a lack of financial resources means that regional health departments must improvise ways to acquire ambulance services. Some health departments have outsourced from organizations such as the Red Cross. Still, there are concerns about the cost-effectiveness of this strategy because the same departments must finance recurrent costs (e.g. driver allowances and payment of paramedics). Lack of emergency transport represents a broader lack of investment in primary health facilities and ultimately requires planning and political goodwill. Kenya has targeted increasing investment in emergency medical services, including the distribution of ambulances, by at least 50% by 2025 [21]. This goal is a step in the right direction but must be matched by sound implementation.

#### Drivers of parallel coordination

Parallel coordination was influenced by heavy workloads and resultant ineffective task shifting within facilities. Heavy workloads have been linked to poor quality [22], delay in induction of labour, and increased caesarean Sections [23], among others. In addition to workloads and understaffing, failure of community follow up influenced parallel coordination. Most nurses in Kenya are trained as registered community health nurses with responsibilities within the community. However, it appears that nurses working in peripheral facilities do not feel this is part of their work description. Lay health workers (community health volunteers) have been home visiting recently. However, a lack of incentivization seems to discourage them. The consequence seems to be that high-risk mothers are not followed up within the community.

#### Access

Implementing simple strategies like toll-free hotlines for pregnant mothers improves continuity of care by maintaining contact between mothers and health providers. This accommodation, driven by local facility staff, is part of a broader package of information and communication technologies. Out-of-pocket payments deter mothers from seeking outpatient care for complications, resulting in emergencies at term. Despite Kenya’s free maternal health policy, high-risk women still face significant out-of-pocket expenses, acting as a disincentive. Near-miss survivors commonly used alternative referral networks outside formal health system pathways. Bypassing non-functional or poorly functional facilities might be associated with better survival odds, aligning with previous qualitative studies on referral experiences [13, 24, 25].

### Strengths and limitations

The study used multiple data collection methods; a strength associated with multimethod case study approaches. However, the researcher is aware of several limitations that may affect the interpretation of results. First, midwives from the referral facility (MTRH) were not part of the focus groups interviewed. It is possible that the researcher would have obtained richer information if he had included this group. Furthermore, the study only focused on public facilities. Private and missions run facilities may have different experiences regarding the topic. Some limitations may be associated with data collection approaches. For example, the researcher cannot rule out impartiality during observation. Also, the sheer volume of documentation required during observation means there is a potential to miss out on some crucial observations. Finally, some participants may not have participated fully during focus group interviews.

### Implications for research, policy, and practice

Enhancing social competence and skills among health professionals working with pregnant women is vital for interpersonal continuity. Scaling up and replicating respectful maternity care studies and encouraging innovative access solutions, like telephone hotlines, is crucial. Facilities should have more flexibility, and efforts should focus on fostering professional relationships between midwives at different healthcare levels to improve sequential coordination. Reducing out-of-pocket payments for specialized care in referral facilities, possibly through conditional cash transfers, should be prioritized. Further research is needed to explore why women don’t attend appointments at higher-level facilities. Lastly, reducing workloads and increasing staffing can positively impact parallel coordination.

## Conclusion

This research explores the perspectives of near-miss survivors and healthcare professionals on care practices that contribute to obstetric near misses. To promote continuity in primary health care for pregnant women, there is a need to focus on social competencies and skills among health care professionals providing obstetric care, encourage accommodation strategies in facilities, reduce or eliminate out-of-pocket payments for referred women, address professional conflicts among midwives at different levels in the referral pathway and address workloads and task shifting in primary care facilities.

## Supplementary Information

Supplementary Material 1.

## Data Availability

The data that support the findings of this study are available on request from the corresponding author [S.M]. The data are not publicly available due to policies from Moi Teaching and Referral Hospital regarding sharing of patient data.

## Abbreviations

ANC: Antenatal Care
CT scan: Computed Tomography scan
CWC: Child Welfare Clinic
EMOC: Emergency Obstetric Care
FGD: Focus Group Discussion
HCP: Health Care Provider
HCPs: Health Care Providers
ICU: Intensive Care Unit
MTRH: Moi Teaching Referral Hospital
PPH: Postpartum Hemorrhage
RH-A: Referral Hospital A
RH-B: Referral Hospital B
SDGs: Sustainable Development Goals
UGDH: Uasin Gishu District Hospital
USD: United States Dollar
VE: Vaginal Examination
WHO: World Health Organization
