# Supplementary material for: “Attitude is the fifth delay”: perspectives of obstetric near-miss survivors and health care professionals on continuity and coordination of maternal care
Source: BMC Health Serv Res. 2025 Feb 19;25:276. doi: 10.1186/s12913-025-12341-4 (PMC11837310; doi:10.1186/s12913-025-12341-4)
Supplement: Supplementary file 1 — Supplementary Material 1. [file 12913_2025_12341_MOESM1_ESM.docx]

Interview guide for the near miss survivors

*These interview guide questions will be refined after phase one of the study after identifying the determinants

**Objective: To explore the perspectives of near miss survivors on activities that influenced coordination and continuity of care processes leading to obstetric near miss cases.**

- ***Tell me about yourself.***

Probes:Age, Parity, Gravidity, where you live, Marital status, Employment status,

- ***Let’s talk about your health before pregnancy***

Probes: chronic conditions, preconceptual care

- ***Let’s talk about your journey during pregnancy***

Probes: When you first attended the ANC clinic

- Continuity of care (Interpersonal; Longitudinal; Managerial; Informational)
- Care coordination (sequential; parallel)
- ***Let’s talk about your journey during birth***

Probes: Where did you deliver your baby?

- Continuity of care (Interpersonal; Longitudinal; Managerial; Informational)
- Care coordination (sequential; parallel)
- ***Let’s talk about your journey after birth of the baby first 42 days***

Probes: When did you deliver your baby?

- Continuity of care (Interpersonal; Longitudinal; Managerial; Informational)
- Care coordination (sequential; parallel)
- **Let’s talk about the near miss incident**
  - What happened
  - Where it happened
  - How do you feel about it?

Observation of activities influencing care coordination and continuity

**Objective Four Observe the activities that influenced the coordination and continuity of care leading to obstetric near miss cases**

| Observer | |
| --- | --- |
| ITEM | Observation |
| Health facility |  |
| Services offered |  |
| No of midwives |  |
| No of doctors |  |
| Date: | |
| Activity 1: Care coordination | Behaviour/observation |
| - Sequential |  |
| - Parallel |  |
| Activity 2: Continuity of care |  |
| - Interpersonal |  |
| - Longitudinal |  |
| - Managerial |  |
| - Informational |  |
| Tools used |  |
| Workflow |  |
| Division of labour |  |
| Work processes |  |
| Team / groups |  |

Interview guide for FGDs

*These interview guide questions will be refined after phase one of the study after determining the prevalence and determinants

*Objective Five: To explore the perspectives of health care professionals on activities that influenced coordination and continuity of care processes leading to obstetric near miss cases*

- *Let us introduce ourselves*

*Probe: Name, age, profession, years of experience*

- *Let us talk about near miss cases*
- Occurrence
- Feelings
- *Let’s talk about Care coordination within and between health care facilities*
- Sequential
- Parallel
- *Let us talk about continuity of care*
- interpersonal.
- Longitudinal.
- Managerial.
- Informational
